# Early detection of retinal and choroidal microvascular impairments in diabetic patients with myopia

**DOI:** 10.3389/fcell.2025.1609928

**Published:** 2025-05-26

**Authors:** Yufei Wu, Jiahui Jiang, Xiaoyu Deng, Xixi Zhang, Jinger Lu, Zian Xu, Yitian Zhao, Zai-Long Chi, Qinkang Lu

**Affiliations:** ^1^ Ophthalmology Center, The Affiliated Peoples Hospital of Ningbo University, Ningbo, Zhejiang, China; ^2^ School of Ophthalmology and Optometry, Wenzhou Medical University, Wenzhou, Zhejiang, China; ^3^ Laboratory of Advanced Theranostic Materials and Technology, Ningbo Institute of Materials Technology and Engineering, Chinese Academy of Sciences, Ningbo, China

**Keywords:** retina, choroid, microvasculature, optical coherence tomography angiography, diabetes, myopia

## Abstract

**Purpose:**

To evaluate and quantify diabetes-related retinal and choroid perfusion changes in individuals with and without high myopia and explore their associations with diabetes risk factors.

**Methods:**

Diabetic patients [n = 133; 43 without diabetic retinopathy in group DM; 48 non-proliferative diabetic retinopathies in group DR; 42 without DR but with high myopia in group HM] underwent ophthalmological and endocrinological examinations. Swept-source optical coherence tomography angiography (SS-OCTA) was used to image the retinal vessel density (RVD), retinal thickness (RT), choroidal thickness (CT), choriocapillaris vessel perfusion (CPV) and choroidal vascularity index (CVI). Automatic segmentation of retinal and choroidal layers was performed using a deep learning-based U-Net architecture. A ResNet-50 convolutional neural network was further applied to analyze vascular density patterns and assist in DR grading. Univariate and multiple linear regression analyses explored the associations between perfusion and risk factors.

**Results:**

The inner ring retinal vessel density and CVI in all areas were significantly different between groups (*P* < 0.05); CPV was not significantly changed except for the inferotemporal area among the groups. CT was decreased in all areas between groups (*P* < 0.05). The visual impairments in HM group was more obvious correlation with the retinal and choroidal structural changes. The AI-driven analysis revealed that decreased CVI and CT were significantly associated with age and spherical equivalent (SE), highlighting the utility of automated algorithms in identifying early microvascular impairments.

**Conclusion:**

Diabetic patients with high myopia exhibited significantly lower CVI compared to those with diabetic retinopathy, indicating that CVI monitoring could facilitate risk stratification of diabetic retinopathy progression. The integration of SS-OCTA with artificial intelligence-enhanced segmentation and vascular analysis provides a refined method for early detection of retinal and choroidal microvascular impairments in diabetic populations.

## Introduction

Diabetes has become one of the most important chronic diseases threatening human health worldwide due to its high incidence, rapid growth, and serious harm (Saeedi et al., 2019). Approximately 30%–40% of diabetic patients will develop at least one complication within 10 years of disease onset (Liew et al., 2014). Diabetic retinopathy (DR) ranks first among retinal vascular diseases that lead to blindness in working-age populations (Jee et al., 2013). DR can be asymptomatic and presents no obvious clinical symptoms in early stages, a complex disease with unclear pathogenesis and a high risk for disability and blindness (Hu et al., 2012).

Although DR is a serious and irreversible cause of blindness, it is also preventable. In the early stages of DR, improving microcirculation and controlling blood glucose through treatment can help slow the progression of the disease, preserving the patient’s vision and reducing the risk of blindness (Li et al., 2021a). Among the known risk factors for DR, diabetes severity and blood glucose control are the most significant (Thomas et al., 2015; Wat et al., 2016). However, some patients may develop DR even if their blood glucose is under good control and the duration of diabetes is relatively short (Silva et al., 2018). In contrast, some patients with poor blood sugar control and a longer duration of diabetes may not develop DR. It is especially important to research factors related to the progression of DR (Li et al., 2021a). Therefore, exploring the factors related to the progression of DR in diabetic patients will enhance the understanding of the mechanism of DR.

The global prevalence of myopia is increasing, and it is projected to affect 50% of the global population by the year 2050 (Holden et al., 2016). Diabetes and myopia have indeed become global health issues, and they are expected to continue to increase in the future (Yuan et al., 2022). Both diseases can lead to vision impairment and can interact with each other (Su et al., 2023). Previous studies have revealed that diabetic patients with myopia have a lower probability of developing DR than those without myopia (Wang et al., 2016; Lin et al., 2020; Yuan et al., 2022). It is believed that myopia may serve as a protective factor against the onset and progression of DR (Man et al., 2019; Lin et al., 2020). an increased axial length (AL) plays a critical role in this protective effect (Dogru et al., 1998; Wang et al., 2016). However, this speculation is still controversial, and some studies suggest that there is no correlation between myopia and DR (Xie et al., 2008; Ganesan et al., 2012). This study hypothesizes that high myopia may modify the patterns of microvascular damage in diabetic patients through structural changes in the choroid. The primary objective was to evaluate retinal and choroidal perfusion changes in diabetic patients with high myopia using SS-OCTA and AI-driven analysis, aiming to identify early biomarkers for DR risk stratification.

## Materials and methods

### Study design and participants

This cross-sectional study was approved by The Affiliated People’s Hospital of Ningbo University ethics committee, which adheres to the tenets of the Declaration of Helsinki. Consent was obtained from all participants prior to participation.

### Subjects

This study was conducted at The Affiliated People’s Hospital of Ningbo University in Zhejiang, China, and we recruited 133 participants with type-2 diabetes mellitus (DM) from April 2023 to June 2023 for this study, including 48 females and 85 males with a mean age of 61 years (range: 52–70 years). As a rule, we selected the right eye of participants, except for those in the DR and HM groups who only had DR or HM on the left eye. Most of the patients with type-2 DM were diagnosed by an endocrine specialist (JL) from the endocrinology department of the Affiliated People’s Hospital of Ningbo University. The patients with moderate to severe NPDR were from the ophthalmology department. The participants were divided into three groups according to the presence of DR and high myopia: diabetes without DR and high myopia in group DM, nonproliferative diabetic retinopathy in group DR, and diabetes without DR but with high myopia in group HM. High myopia was defined as having an AL greater than or equal to 25.5 mm without pathologic myopia (PM) (Flitcroft et al., 2019). The PM includes chorioretinal atrophy, patchy chorioretinal atrophy, and macular atrophy according to the International Meta-Analysis for Pathologic Myopia (META-PM) classification system (Ohno-Matsui et al., 2015). The exclusion criteria were as follows: 1) intraocular pressure (IOP) > 21 mmHg; 2) previous diagnosis of retinal or choroidal diseases except DR, such as glaucoma, uveitis, retinal vascular occlusion, or age-related macular degeneration (AMD); 3) history of retinal or intraocular surgery; 4) opacity of refractive medium that may affect anterior and posterior section imaging; and 5) systemic disease other than diabetes and hypertension, such as tumor, cerebral infarction, stroke, or mental illness. All subjects underwent comprehensive ophthalmologic examinations, including the best corrected visual acuity (BCVA) test, intraocular pressure (IOP), refraction, spherical equivalent (SE), AL and slit-lamp biomicroscopy. All participants completed a demographic and clinical characteristics questionnaire that included their age, sex, blood pressure, height, weight, past systemic and ocular medical history, duration of diabetes, type of diabetes and current medication status. Laboratory serum biochemical indicators of all the participants were also collected on the same day, including HbA1c level, blood glucose (BG), total cholesterol (T-CHOL), triglyceride (TG), high-density lipoprotein cholesterol (HDL-C), low-density lipoprotein cholesterol (LDL-C) and serum creatinine (SCR).

### Fundus photography and staging of DR

**FIGURE 1:**
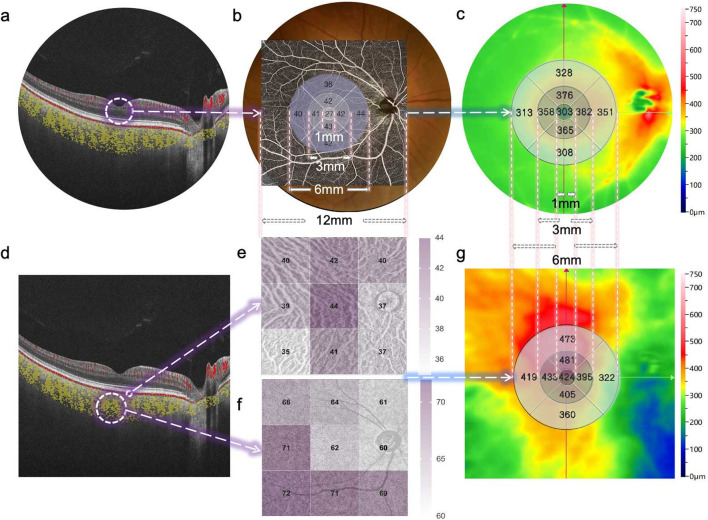
Representative SS-OCTA images obtained from a diabetic patient’s eye (12 × 12 mm area). **(a)** B-scan image of retinal layers. **(b)** Fundus photography of the retina and its corresponding macula center (CM) (1 mm diameter) and fan-shaped areas of 1–3 mm, 3–6 mm, and 6–12 mm on OCTA, as well as the quantified topography of the RVD in a total area of 6 × 6 mm. **(c)** The quantified topography of RT in a total area of 6 × 6 mm. **(d)** B-scan image of choriocapillary and ChV layers. **(e)** The quantified topography of CPV in nine subfields (superotemporal, temporal, inferotemporal, superior, central, inferior, superonasal, nasal, inferonasal) of the en-face image. **(f)** The quantified topography of CVI in nine subfields of en-face image. **(g)** The quantified topography of CT in a total area of 6 × 6 mm.

All participants underwent fundus photography centered around the macular fovea by a digital fundus camera (Canon CR-2 AF; Tokyo, Japan) (Figure 1b). Two ophthalmologists (YW and XD) graded DR according to the international clinical DR proposed by the Global DR Project Group (Wilkinson et al., 2003) that categorizes the severity of DR as follows: 0: no apparent retinopathy (NDR), 1: mild nonproliferative DR (NPDR), 2: moderate NPDR, 3: severe NPDR and 4: proliferative DR (PDR). If there was a discrepancy in grading, it was graded by another senior ophthalmologist.

### Swept source optical coherence tomography angiography (SS-OCTA) imaging and analysis

**FIGURE 2:**
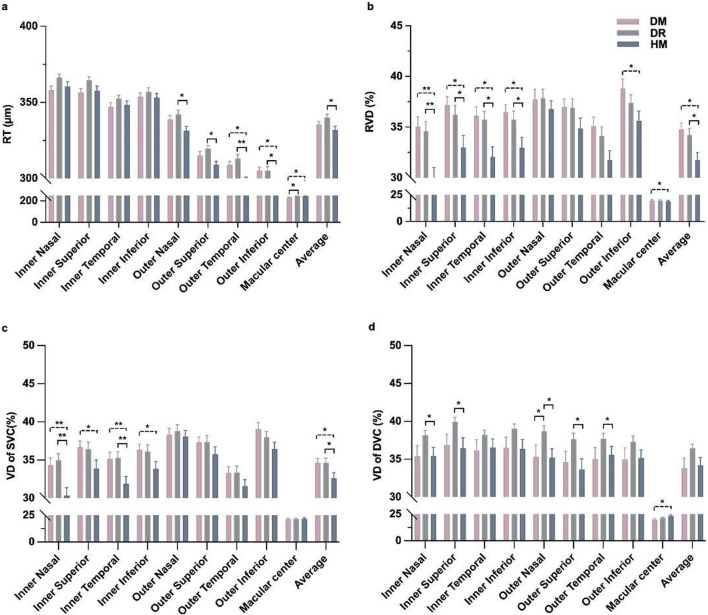
Comparison*s* of retinal thickness and vessel density in OCT-A images among the three groups around the macular area. **(a)** Thickness of the whole retina. **(b)** The vascular density of the whole retina. **(c)** The vascular density of the superficial capillary plexus. **(d)** The vascular density of the deep capillary plexus. *P < 0.05, **P < 0.001.

One skilled ophthalmologist (JJ) performed the retinal and choroidal images with the 400 kHz SS-OCTA instrument (BM400K BMizar, TowardPi Medical Technology Co., Ltd., Beijing, China) between 8 AM and 12 AM every day. It provides a transverse resolution of 10 µm and an axial optical resolution of 3.8 µm. To eliminate eye artifacts, it was also equipped with an eye tracking tool based on an integrated confocal scanning laser ophthalmoscope. The scan mode was 12 × 12 mm centered around the central macular concavity, and both b-scan and en-face images were obtained and stored for analysis. The algorithm applied to detect motion signals is called higher-order moments amplitude decorrelation angiography (HMADA). It is an effective visualization technique of both large blood vessels and the capillary network in the retinal and choroidal circulations, by capturing higher order statistical signals in OCTA data. With Al technology, each layer including BM and choroid-sclera interface is able to be recognized. The *en face* OCT and OCTA images of retinal and choroidal segmentation (automatic or manual) are well visualized. The area indicated by the circle was the retinal vessel density (RVD), which was defined as the capillary vascular density between the inner limiting membrane and outer plexiform layer (Figure 1a). The OCT-A mode (6 × 6 mm area) was also used to obtain macular microvascular images (Figures 2b,c) of the superficial and deep retinal vessel densities (SVD and DVD), and the retinal thickness were computed automatically for nine subfields (superotemporal, temporal, inferotemporal, superior, central, inferior, superonasal, nasal, inferonasal). The yellow area indicated by the circle is the choroidal vessel (ChV) layer obtained by OCT-A mode (12 × 12 mm area). The ChV was divided into the choriocapillaris layer (from 29 µm posterior to the retinal pigment epithelium) and the medium- and large-vessel layer (Figures 1d–f). The choroidal vascularity index (CVI) was defined as the ratio of the choroidal vascular luminal volume to the total choroidal volume, which reflects the volumetric choroidal vascular density (Yang et al., 2020). The choroidal thickness (CT) was divided into three regions and four quadrants (temporal, nasal, superior and inferior).

### Statistical analysis

All continuous variables were analyzed with SPSS software (version 23.0; SPSS, Inc., Chicago, IL, United States) and expressed as the means ± standard deviations. Bonferroni correction was applied for multiple group comparisons, with the significance threshold adjusted to P < 0.017 (0.05/3). The Chi-square test was used to determine gender differences within each of the three groups. One-way analysis of variance (one-way ANOVA) was used to test for differences among the three groups, followed by *post hoc* pairwise analyses with Bonferroni-adjusted P values. Post hoc tests were used for group pairs. We used Pearson’s correlation to analyze relationships between visual function and retinal and choroid microstructure parameters. Generalized estimating equations (GEEs) were used to quantify correlations between axial magnification and choroid parameters. The correlations between influencing factors [sex, age, body mass index (BMI), IOP, AL, HbA1c, duration of disease] and choroid parameters [choriocapillaris vessel perfusion (CPV), CVI, CT] were tested by univariate correlation and multiple linear regression analyses. Variables with p < 0.1 in univariate analysis plus clinically relevant factors.

## Results

### Patient characteristics

A total of 133 eyes from 133 participants with DM were assessed in this study, including 43 eyes with NDR, 48 eyes with NPDR, and 42 eyes with HM. There were no significant differences in any other baseline characteristics among the three groups, such as age, sex, BMI, MAP, BCVA and IOP (*P* = 0.196–0.823). The HbA1c (*P* = 0.479), BG (*P* = 0.712), TG (*P* = 0.534), T-CHOL (*P* = 0.416), HDL-C (*P* = 0.937), LDL-C (*P* = 0.350) and SCR (*P* = 0.903) among the three groups also showed no significant differences. The DR group had a longer duration of diabetes, and the HM group had a lower SE and longer AL than the other groups (*P* < 0.001; Table 1).

**TABLE 1:** Demographics and clinical characteristics of subjects.

Parameters	Group			*P*
	DM, n = 43	DR, n = 48	HM, n = 42	
Age, y	62.86 ± 6.65	61.56 ± 8.72	60.52 ± 8.03	0.394^a^
Sex, M/F	27 : 16	35 : 13	23 : 19	0.196^b^
BMI, kg/m^2^	24.47 ± 3.65	24.89 ± 3.04	24.61 ± 3.15	0.823^a^
MAP, mmHg	99.96 ± 10.15	97.53 ± 8.44	96.58 ± 9.35	0.226^a^
SE, diopter	0.50 ± 0.87	0.05 ± 1.03	−4.89 ± 1.97	**< 0.001** ^a^
BCVA, log Mar	0.23 ± 0.17	0.28 ± 0.18	0.29 ± 0.20	0.211^a^
AL, mm	22.93 ± 0.73	23.32 ± 0.69	25.91 ± 1.37	**< 0.001** ^a^
IOP, mmHg	14.03 ± 2.95	15.01 ± 3.14	15.04 ± 3.23	0.231^a^
Duration, y	6.93 ± 5.77	11.79 ± 6.93	6.71 ± 5.84	**< 0.001** ^a^
HbA1c, %	6.91 ± 1.07	7.22 ± 1.31	7.10 ± 1.21	0.479^a^
BG, mmol/L	7.51 ± 1.38	7.85 ± 2.39	7.82 ± 2.47	0.712^a^
TG, mmol/L	1.66 ± 1.05	1.96 ± 1.52	1.82 ± 1.22	0.534^a^
T-CHOL, mmol/L	4.76 ± 0.98	5.04 ± 1.08	4.79 ± 1.25	0.416^a^
HDL-C, mmol/L	1.30 ± 0.29	1.28 ± 0.27	1.30 ± 0.34	0.937^a^
LDL-C, mmol/L	2.45 ± 0.70	2.69 ± 0.83	2.52 ± 0.84	0.350^a^
SCR, μmol/L	73.81 ± 35.37	72.50 ± 24.67	71.12 ± 20.30	0.903^a^

Values for continuous variables are means ± standard deviations for all subjects in each group.

Boldface values indicate statistically significant differences at *P* < 0.05.

^a^ One-way ANOVA, followed by the *post hoc* LSD, test.

^b^ χ2 test.

DM, diabetic patients without diabetic retinopathy; DR, Non-proliferative diabetic retinopathy; HM, diabetic patients with high myopia; M, male; F, female; SE, spherical equivalent; BCVA, best-corrected visual acuity; AL, axial length; IOP, intraocular pressure; BG, blood glucose; TG, triglyceride; T-CHOL, total cholesterol; HDL-C, High-density lipoprotein cholesterol; LDL-C, Low-density lipoprotein cholesterol; SCR, serum creatinine.

MAP, Diastolic pressure +1/3 pulse pressure difference.

BMI, Weight/(Height)^2^.

### Retinal thickness and vessel perfusion parameters

The inner nasal and superior grids of the RT were significantly different between the DM and DR groups (*P* = 0.025 and, 0.028, respectively; Table 2). There were differences in the outer temporal and inferior grids of RT between the DM and HM groups (*P* = 0.004 and, 0.020, respectively; Table 2). The RT of the outer grids in the HM group was thinner than that in the DR group. (*P* = 0.001 to 0.016; Table 2; Figure 2a). There were differences in the RVD in the inner regions among the groups (*P* = 0.001 to 0.026; Supplementary Table S1; Figure 2b). The SVD in the inner grids of the HM group was lower than that of the other groups (*P* = 0.001 to 0.044; Figure 2c). The DVD was increased in some grids of the DR group compared with the others (*P* = 0.013 to 0.042; Figure 2d).

**TABLE 2:** Comparison of CT and RT value among the three groups.

		Group			*P*1^a^	*P*2^a^	*P*3^a^
		DM	DR	HM			
RT, μm Mean ± SD							
Average		335.47 ± 14.13	340.13 ± 13.92	331.95 ± 15.03	0.124	0.261	**0.008**
Macular center		234.19 ± 14.89	245.08 ± 25.02	246.24 ± 20.74	**0.014**	**0.009**	0.793
Inner	Nasal	358.14 ± 17.28	366.38 ± 15.13	360.48 ± 19.38	**0.025**	0.534	0.108
	Superior	356.49 ± 17.06	364.65 ± 15.12	357.55 ± 20.30	**0.028**	0.781	0.057
	Temporal	347.19 ± 17.09	352.58 ± 15.37	348.40 ± 16.13	0.115	0.729	0.224
	Inferior	353.72 ± 17.03	357.06 ± 17.14	353.17 ± 17.30	0.355	0.882	0.284
Outer	Nasal	338.93 ± 16.46	342.29 ± 19.01	331.50 ± 18.33	0.376	0.059	**0.005**
	Superior	315.12 ± 18.74	319.63 ± 14.36	308.98 ± 14.45	0.180	0.078	**0.002**
	Temple	308.98 ± 14.74	313.19 ± 18.75	298.90 ± 13.32	0.210	**0.004**	**< 0.001**
	Inferior	305.16 ± 15.39	305.25 ± 17.62	296.64 ± 16.71	0.980	**0.020**	**0.016**
CT, μm Mean ± SD							
Average		274.19 ± 80.50	220.04 ± 69.68	177.56 ± 58.37	**< 0.001**	**< 0.001**	**0.005**
Macular center		288.19 ± 86.43	239.15 ± 88.18	191.24 ± 65.85	**0.005**	**< 0.001**	**0.006**
Inner	Nasal	275.30 ± 86.71	219.17 ± 83.62	167.21 ± 59.07	**0.001**	**< 0.001**	**0.002**
	Superior	297.35 ± 87.44	242.40 ± 76.27	193.07 ± 70.59	**0.001**	**< 0.001**	**0.003**
	Temporal	288.70 ± 79.56	238.94 ± 75.87	196.40 ± 68.81	**0.002**	**< 0.001**	**0.008**
	Inferior	282.14 ± 89.02	227.13 ± 90.19	180.21 ± 69.28	**0.002**	**< 0.001**	**0.009**
Outer	Nasal	232.95 ± 87.50	171.33 ± 73.25	133.55 ± 55.44	**< 0.001**	**< 0.001**	**0.016**
	Superior	285.70 ± 91.94	235.79 ± 66.16	195.50 ± 61.92	**0.002**	**< 0.001**	**0.011**
	Temple	263.30 ± 81.47	221.63 ± 62.49	187.29 ± 65.83	**0.005**	**< 0.001**	**0.022**
	Inferior	268.09 ± 93.26	203.96 ± 77.31	167.21 ± 66.93	**< 0.001**	**< 0.001**	**0.031**

Values for continuous variables are means ± standard deviations for all subjects in each group.

Boldface values indicate statistically significant differences at *P* < 0.05.

*P*1: comparison between the group DM, and DR; *P*2: comparison between the group DM, and HM; *P*3: comparison between the group DR, and HM.

^a^ One-way ANOVA, followed by the *post hoc* LSD, test.

DM, diabetic patients without diabetic retinopathy; DR, Non-proliferative diabetic retinopathy; HM, Diabetic patients with high myopia CT, choroidal thickness.

RT, retinal thickness.

### Choroidal thickness and vessel perfusion parameters

**FIGURE 3:**
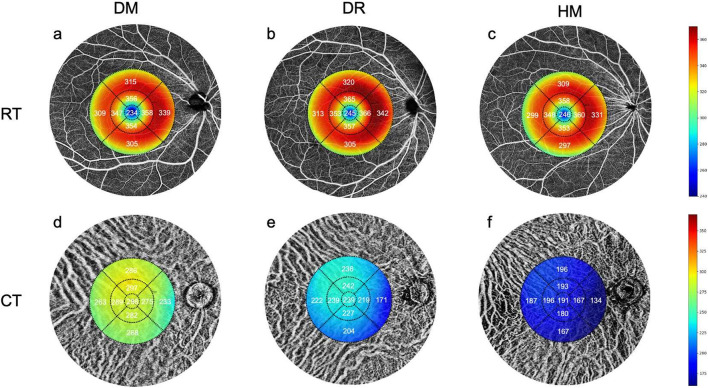
Comparisons of the retinal and choroidal thickness in OCT-A images among the three groups. **(a,d)** The mean values of RT and CT in the DM group. **(b,e)** The mean values of RT and CT in the DR group. **(c,f)** The mean values of RT and CT in the HM group.

In one-way ANOVA, the CT of each grid session in all areas significantly differed between groups (Table 2; Figures 3d–f). In the *post hoc* pairwise analysis, the CT in HM eyes was significantly thinner than that in the DM and DR groups (*P* = 0.001 to 0.031; Table 2). The CT in the DR group was significantly thinner than that in the DM group (*P* = 0.001 to 0.005; Table 2).

**FIGURE 4:**
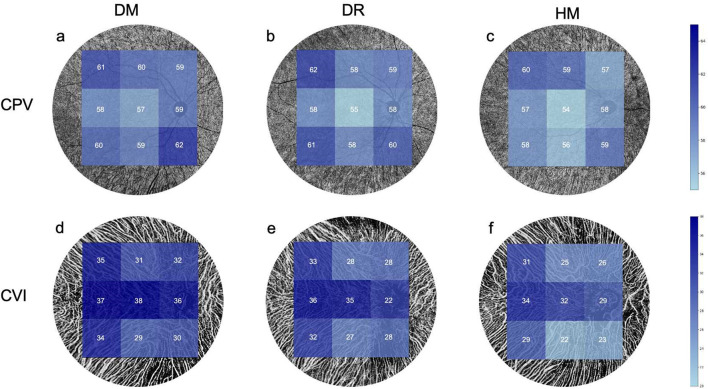
Comparisons of the choroidal perfusion in OCT-A images among the three groups. **(a,d)** The mean value of the CPV and CVI in the DM group. **(b,e)** The mean value of the CPV and CVI in the DR group. **(c,f)** The mean value of the CPV and VI in the HM group.

There was no difference in the CPV between the groups, except for the inferotemporal area (*P* = 0.006; Table 3; Figures 4a–c). The CVI of the NPDR group was lower in the superotemporal, superior, central, superonasal and nasal regions and the average sector when compared to the DM group (*P* = 0.042, 0.014, 0.038, 0.006, 0.009 and, 0.006, respectively; Table 3; Figures 4d–f). All regions of the CVI in the HM group were significantly lower than those in the DM group (*P* < 0.001; Table 3). Compared to the group DR, the group HM had a significantly lower CVI in most sectors (*P* = 0.002 to 0.028; Table 3), except for the superotemporal, superior and superonasal sectors (*P* = 0.121, 0.179 and 0.142, respectively; Table 3). The patients in group DR showed significantly reduced CVI in most sectors when compared to the DM group (*P* = 0.006 to 0.042; Table 3), except for the temporal, inferotemporal, inferior and inferonasal sectors (*P* = 0.061, 0.057, 0.100 and 0.214, respectively; Table 3).

**TABLE 3:** Comparison of CPV and CVI value among the three groups.

	Group			*P*1^a^	*P*2^a^	*P*3^a^
	DM	DR	HM			
CPV, %Mean ± SD						
Average	59.49 ± 10.02	58.93 ± 9.75	57.78 ± 10.96	0.792	0.441	0.596
superotemporal	61.12 ± 10.27	61.56 ± 10.73	60.24 ± 12.12	0.848	0.715	0.571
temporal	58.26 ± 10.70	58.23 ± 10.38	57.83 ± 12.74	0.991	0.863	0.868
inferotemporal	59.91 ± 12.52	61.04 ± 11.12	58.10 ± 12.11	**0.006**	0.484	0.243
superior	59.65 ± 11.72	58.44 ± 9.89	59.71 ± 11.43	0.600	0.979	0.584
central	57.12 ± 9.49	55.04 ± 9.38	54.26 ± 13.14	0.360	0.223	0.732
inferior	58.79 ± 8.70	58.31 ± 10.39	56.10 ± 11.69	0.826	0.231	0.311
superonasal	59.21 ± 12.98	59.06 ± 11.68	57.00 ± 11.85	0.954	0.404	0.424
nasal	59.26 ± 11.21	58.23 ± 10.34	57.48 ± 11.46	0.657	0.457	0.746
inferonasal	62.14 ± 10.86	60.42 ± 12.38	59.29 ± 13.67	0.508	0.289	0.665
CVI, %Mean ± SD						
Average	33.85 ± 4.66	31.23 ± 4.01	28.34 ± 4.75	**0.006**	**< 0.001**	**0.003**
superotemporal	35.49 ± 4.28	33.27 ± 5.45	31.57 ± 5.58	**0.042**	**< 0.001**	0.121
temporal	37.60 ± 4.04	35.92 ± 3.76	33.88 ± 4.93	0.061	**< 0.001**	**0.025**
inferotemporal	34.56 ± 5.08	32.35 ± 5.03	29.79 ± 6.28	0.057	**< 0.001**	**0.028**
superior	31.02 ± 6.23	27.73 ± 6.71	25.93 ± 5.89	**0.014**	**< 0.001**	0.179
central	37.86 ± 4.88	35.54 ± 4.69	31.98 ± 6.21	**0.038**	**< 0.001**	**0.002**
inferior	29.63 ± 7.85	26.94 ± 7.67	22.40 ± 7.72	0.100	**< 0.001**	**0.006**
superonasal	32.07 ± 6.54	28.42 ± 5.78	26.48 ± 6.36	**0.006**	**< 0.001**	0.142
nasal	35.98 ± 5.15	32.63 ± 5.44	29.60 ± 7.39	**0.009**	**< 0.001**	**0.019**
inferonasal	30.47 ± 8.34	28.25 ± 8.91	23.45 ± 8.00	0.214	**< 0.001**	**0.008**

Values for continuous variables are means ± standard deviations for all subjects in each group.

Boldface values indicate statistically significant differences at *P* < 0.05.

*P*1: comparison between the group DM, and DR; *P*2: comparison between the group DM, and HM; *P*3: comparison between the group DR, and HM.

^a^ One-way ANOVA, followed by the *post hoc* LSD, test.

DM, diabetic patients without diabetic retinopathy; DR, Non-proliferative diabetic retinopathy; HM, Diabetic patients with high myopia CT, choroidal thickness.

CPV, choriocapillaris vessel perfusion.

CVI, choroidal vessel perfusion index.

### Relationships among the choroidal parameters, visual function, and risk factors

We analyzed the correlation of choroid structural and perfusion parameters with BCVA and risk factors, including age, BMI, MAP, SE, AL, IOP, duration and HbA1c. In the DM group, the BCVA was negatively correlated with the inner and outer superior RVD and superonasal CPV (r = −0.313 to −0.334, *P* < 0.05; Table 4). In the HM group, we found that there was a significant correlation between BCVA and outer superior SVC and DVC, outer superior RVD, almost all grid CPV and supertemporal CVI (r = −0.306 to −0.460, *P* < 0.05; Table 4).

**TABLE 4:** The correlation between OCTA value and visual functions among the three groups.

		Group		
		DM(r, p)	DR(r, p)	HM(r, p)
SVC	Outer Superior	−0.319 (**0.037**)	−0.038 (0.797)	−0.460 (**0.002**)
DVC	Outer Superior	−0.093 (0.553)	−0.124 (0.401)	−0.393 (**0.010**)
RVD	Inner Superior	−0.313 (**0.041**)	−0.070 (0.635)	−0.050 (0.752)
	Outer Superior	−0.332 (**0.030**)	−0.017 (0.910)	−0.417 (**0.006**)
	Superotemporal	−0.021 (0.896)	−0.209 (0.154)	−0.376 (**0.014**)
	Temporal	−0.013 (0.935)	−0.123 (0.406)	−0.412 (**0.007**)
CPV	Superior	−0.040 (0.799)	0.294 (**0.042**)	−0.306 (**0.048**)
	Central	0.018 (0.910)	−0.156 (0.290)	−0.371 (**0.016**)
	Superonasal	−0.334 (**0.028**)	−0.156 (0.290)	−0.076 (0.633)
CVI	Superonasal	−0.051 (0.744)	−0.048 (0.744)	−0.361 (**0.019**)

The correlation coefficient is displayed outside parentheses. The *P* value is displayed within the parentheses.

Boldface values indicate statistically significant differences at *P* < 0.05.

DM, diabetic patients without diabetic retinopathy; DR, Non-proliferative diabetic retinopathy; HM, Diabetic patients with high myopia CT, choroidal thickness.

SVC, superficial vascular complex.

DVC, deep vascular complex.

RVD, vascular density of retinal.

CPV, choriocapillaris vessel perfusion.

CVI, choroidal vessel perfusion index.

**FIGURE 5:**
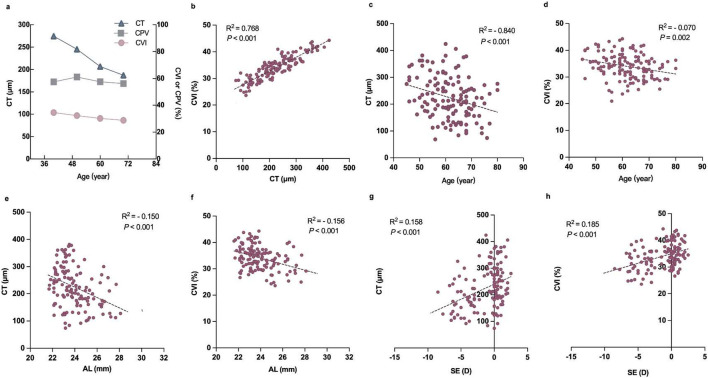
Correlation between the relevant factors and choroidal parameters in diabetic subjects. **(a)** Plot showing the trend of CT, CPV, and CVI changes with age. **(b)** Correlation between CT and CVI. **(c)** Correlation between age and CT. **(d)** Correlation between age and CVI. **(e)** Correlation between AL and CT. **(f)** Correlation between AL and CVI. **(g)** Correlation between SE and CT. **(h)** Correlation between SE and CVI.

CT was positively associated with CVI vessel density (R^2^ = 0.768, *P* < 0.001; Figure 5b). The CT and CVI were negatively correlated with age and AL (R^2^ = −0.840, *P* < 0.001; R^2^ = −0.070, *P* = 0.002; R^2^ = −0.510, *P* < 0.001, R^2^ = −0.516, *P* < 0.001, respectively; Figures 5c–f) but positively correlated with SE (R^2^ = 0.158, *P* < 0.001; R^2^ = 0.185, *P* < 0.001; Figures 5g,h). Multiple linear regression analyses showed that age and SE increased the risk for decreased CVI and CT in patients with diabetes (*P* < 0.05; Table 5).

**TABLE 5:** Regression analysis of risk factors for decreased choroidal perfusion in diabetic patients.

		Unstandardized β (standardized β)	*P*	95%CI
CPV	Age	−0.275 (0.113)	**0.015**	−0.496–−0.054
	BMI	0.398 (0.267)	0.135	−0.124–0.921
	MAP	−0.107 (0.092)	0.247	−0.288–0.074
	SE	0.487 (0.627)	0.437	−0.741–1.715
	AL	−0.473 (1.044)	0.650	−2.521–1.574
	IOP	0.006 (0.283)	0.983	−0.549–0.561
	Duration	−0.097 (0.130)	0.456	−0.351–0.158
	HbA1c	−1.889 (1.130)	0.094	−4.103–0.324
CVI	Age	−0.228 (0.048)	**< 0.001**	−0.321–−0.135
	BMI	−0.001 (0.1124)	0.999	−0.220–0.22
	MAP	0.046 (0.039)	0.234	−0.030–0.123
	SE	0.855 (0.264)	**< 0.001**	0.337–1.373
	AL	−0.072 (0.440)	0.870	−0.935–0.791
	IOP	0.095 (0.119)	0.426	−0.139–0.329
	Duration	−0.018 (0.055)	0.736	−0.126–0.089
	HbA1c	0.162 (0.476)	0.734	−0.771–1.095
CT	Age	−3.994 (0.780)	**< 0.001**	−5.522–−2.466
	BMI	−1.192 (1.8422)	0.518	−4.802–2.419
	MAP	0.713 (0.639)	0.264	−0.539–1.965
	SE	12.316 (4.3302)	**0.004**	3.829–20.803
	AL	−3.071 (7.220)	0.671	−17.222–11.079
	IOP	1.246 (1.957)	0.524	−2.590–5.083
	Duration	−0.266 (0.8975)	0.767	−2.025–1.493
	HbA1c	−1.591 (7.806)	0.838	−16.89–13.708

Coefficient is the average increase in CPV, CVI, or CT, for a one unit increase in the covariate (age, AL, or SE) while controlling for other covariates.

Boldface values indicate statistically significant differences at *P* < 0.05.

RVD, vascular density of retinal.

CT, choroidal thickness.

CVI, choroidal vessel perfusion index.

CPV, choriocapillaris vessel perfusion.

## Discussion

In this study, we systematically evaluated retinal and choroidal microvascular alterations in diabetic patients with high myopia using UWF-SS-OCTA combined with artificial intelligence AI algorithms. Several studies have investigated the characteristics of retinal and choroidal changes in myopic diabetic patients (Chao et al., 2016; Man et al., 2019; Lin et al., 2020), but none have been comprehensive. Specifically, recent studies have reported that different phenotypes of macular ischemia might account for different severities of visual function loss (Usman, 2018; Tsai et al., 2023). However, some studies only studied the characteristics of retinal changes, while others only explored the characteristics of choroidal changes without studying the related risk factors. It is unclear whether myopia has a protective effect on DR, as well as its role. According to the present study, diabetic patients with high myopia had decreased retinal structure and choroid perfusion at the early stages, which was especially noticeable in the middle and large vessels of the choroid. Visual impairment was closely related to a decrease in ChV perfusion. The findings of the study will enable further understanding of the pathophysiology of diabetes with myopia and explore the morphological parameters of early visual function impairment.

The interaction between DM and macular microvascular reduction has received a great deal of attention in recent years (Barot et al., 2013; Shin et al., 2019). In our study, the retinal whole vascular density and the superficial vascular density showed a decreasing trend in DR and NDR with HM when compared with the diabetic group, while the deep vascular density was increased in the DR group. Specifically, a study found that vascular changes in eyes with no to early DR were present primarily in the deeper vascular layers, whereas in eyes with advanced DR, the opposite was observed (Ashraf et al., 2020). Another study found that diabetic patients exhibited significantly lower retinal VD in deep vascular complexes than controls. These findings suggest that the deep vascular density is affected differently than the superficial vascular density in DR (Li et al., 2021b). In the current study, patients with DR had a long course of disease, and some had advanced stages. We documented that increased DVD in eyes with NPDR might be a confounding factor for revealing the relationship between DR and degrees of severity. Future studies to explore how different stages of DR are associated with specific layers in the retina and consequently impact visual function are warranted. Indeed, the decrease in RVD was found to be associated with age and duration of diabetes (Thomas et al., 2015; Wat et al., 2016). This suggests that these factors may contribute to the development and progression of DR. These factors can interact with and contribute to changes in retinal vascular density. Regular eye examinations are essential for individuals with diabetes, especially as they age and their duration of diabetes increases. Monitoring both intraocular pressure and retinal vascular density can help healthcare professionals assess the health of the eye and make necessary recommendations for managing and preserving vision in diabetic individuals.

The choroid is a highly vascularized tissue that plays a multifunctional role in the eye (Nickla and Wallman, 2010). Its main function is to supply oxygen and nutrients to the outer retina (Hurley, 2021). The choroid also provides metabolic exchange between the blood and the outer retinal layers, which is essential for the proper functioning of the retina (Linsenmeier and Padnick-Silver, 2000). Previous studies have suggested that the choroid is implicated not only in the pathogenesis of many chorioretinal diseases but also in the progression of DR (Wang et al., 2020) and myopia (Wang Y. et al., 2023) development. Studies have demonstrated that choroidal thinning accompanies the development and progression of myopia (Read et al., 2013; Wang et al., 2020), and a close link was established between eye growth and choroidal thickness changes (Bartol-Puyal et al., 2019). In diabetic patients, choroidal structural changes have been found to correlate with the severity of DR (Wang H. et al., 2023). A study by Wang et al. (Wang H. et al., 2023) used SD-OCTA to show that diabetic patients had a lower choroidal perfusion density than healthy controls. In addition, Wang et al. (Wang et al., 2020) reported decreased choroidal perfusion in diabetic patients without DR using SS-OCTA. However, Dai et al. (Dai et al., 2020) reported that diabetic patients’ choriocapillaris blood FDs were significantly higher than those of controls. Our current study showed that both CT and the CVI decreased significantly with disease severity in individuals with diabetes, and visual impairment was closely related to choroidal perfusion among the groups. Interestingly, CPV did not change significantly in patients with high myopia. Zhao et al. (Zhao et al., 2023) also found no statistically significant difference in CPV between DM patients and a control group. Diabetic choroidopathy (DC) might preferentially affect larger choroidal veins (Haller’s layer) initially before medium (Sattler’s layer) and small (choriocapillaris) arterioles (Foo et al., 2020). It is possible that in diabetic patients with myopia, the occurrence of DR is delayed or reduced because of changes in the structure and perfusion of the retina and choroid, as well as a decrease in tissue oxygen demand.

Data on the relationship between risk factors and the CVI in diabetic populations are still scarce. A longer duration of diabetes has been associated with a decreased CVI of Sattler’s layers and subfoveal choroidal volume, indicating a decline in choroidal perfusion (Foo et al., 2020). Several studies have reported that CT is associated with age and AL (Read et al., 2013; Agrawal et al., 2016; Xiong et al., 2017), and the proportion of CVI has not been consistently demonstrated to change with age and AL. He et al. (Xuan et al., 2023) reported negative correlations between the CVI and age, whereas Zhou et al. (Zhou et al., 2020) found no significant association between the CVI and age in healthy adults by univariable analyses. Furthermore, the significant associations of the CVI with age and AL disappeared when CT was included as an independent variable in a healthy adult population study (Agrawal et al., 2016). The purpose of this study was to examine the choroidal changes in DR and high myopia patients, as well as to investigate the factors influencing choroidal changes. Our results indicated that both CT and the CVI were associated with age, AL and SE in diabetic patients. However, based on the multiple linear regression, after adjusting for all interaction factors, only age and SE were related to CT and CVI. The findings of this study contribute to our understanding of the factors influencing CT and CVI in diabetic patients with high myopia. Nevertheless, more research is needed to confirm these results and explore the underlying mechanisms of the observed relationships. The automated analytical workflow in this study demonstrates that AI-driven OCTA image interpretation significantly improves the detection efficiency of diabetic microvascular damage. By integrating deep learning-extracted features with traditional biometric parameters (e.g., CVI and CT), multimodal risk prediction models could be constructed for future applications. Recent studies indicate that Transformer-based vision models exhibit high sensitivity in identifying early-stage microvascular abnormalities in DR (Schmidt-Erfurth et al., 2018), offering a new direction for developing intelligent decision-making systems to personalize DR screening frequency.

We acknowledge several limitations of the present study. First, our study could not determine causality due to the inherent limitations of cross-sectional studies. The question of whether microvascular alterations in myopic eyes occur before or after DM remains uncertain. Longitudinal analysis should be performed to further understand the relationships. Second, due to the poor cooperation of the diabetic patients, we only selected the choroidal blood supply of the macular area and the peripheral area in the scan mode of 12 × 12 mm area and did not select a larger mode, which may have resulted in a loss of important peripheral lesion information. Third, the exclusion criterion in the current study was relatively stringent, aiming to explore the characteristics of choroidal changes in patients with simple diabetes and patients with myopia. Therefore, patients with other systemic diseases were excluded from the study. Nevertheless, the associations between these variables may still reflect the intrinsic links or underlying mechanisms.

## Conclusion

This study evaluated and quantified diabetes-related retinal and choroid perfusion changes in individuals with and without high myopia. We found that the retina and choroid of diabetic patients with high myopia had a lower vessel density and thinner thickness, even lower than those with DR. The integration of deep learning algorithms for automated OCTA image segmentation and ResNet-50-based vascular analysis significantly enhanced the detection sensitivity of early microvascular alterations, particularly in CVI quantification. Diabetic eyes with high myopia had a lower macular CVI among the three groups, although there were no significant differences in CPV, indicating that AI-assisted monitoring of the CVI could facilitate precise risk stratification of the diabetic population and optimize personalized DR screening strategies. When analyzing choroidal alterations in diabetic eyes, age and spherical equivalent should be considered. Future studies should explore multimodal AI models combining structural and vascular parameters to predict disease progression and guide clinical interventions.

## Data Availability

The original contributions presented in the study are included in the article/Supplementary Material, further inquiries can be directed to the corresponding authors.
