# Supplementary material for: Early detection of retinal and choroidal microvascular impairments in diabetic patients with myopia
Source: Front Cell Dev Biol. 2025 May 26;13:1609928. doi: 10.3389/fcell.2025.1609928 (PMC12146373; doi:10.3389/fcell.2025.1609928)
Supplement: Supplementary file 1 [file Table1.docx]

**TABLE S1** Comparison of VD of SVC, DVC and RVD value among the three groups.

|  | | | **DM** | **DR** | | **HM** | ***P*1*** | | ***P*2*** | ***P*3*** |
| --- | --- | --- | --- | --- | --- | --- | --- | --- | --- | --- |
| **RVD** | |  | | |  | | |  | | |
| Average | | | 34.80±4.02 | 34.23±4.50 | | 31.74±4.84 | 0.548 | | **0.002** | **0.009** |
| Macular center | | | 19.70±6.28 | 19.56±5.68 | | 18.62±5.57 | 0.913 | | 0.397 | 0.447 |
| Inner | Nasal | | 35.05±6.30 | 34.58±6.61 | | 29.95±6.92 | 0.739 | | **0.001** | **0.001** |
|  | Superior | | 37.19±5.36 | 36.23±6.43 | | 33.00±7.60 | 0.485 | | **0.004** | **0.020** |
|  | Temporal | | 36.12±5.86 | 35.73±5.57 | | 32.05±6.42 | 0.757 | | **0.002** | **0.004** |
|  | Inferior | | 36.49±4.82 | 35.73±5.90 | | 32.98±6.51 | 0.533 | | **0.006** | **0.026** |
| Outer | Nasal | | 37.74±6.45 | 37.85±6.17 | | 36.76±5.49 | 0.931 | | 0.456 | 0.395 |
|  | Superior | | 36.98±5.12 | 36.90±6.12 | | 34.88±6.51 | 0.948 | | 0.107 | 0.111 |
|  | Temple | | 35.12±5.72 | 34.13±6.16 | | 31.76±5.97 | 0.430 | | **0.011** | 0.063 |
|  | Inferior | | 38.81±6.04 | 37.40±5.42 | | 35.64±6.03 | 0.248 | | **0.013** | 0.156 |
| **SVC** | | | | | | | | | | |
| Average | | | 34.67±3.68 | 34.64±4.24 | | 32.62±4.67 | 0.975 | | **0.026** | **0.025** |
| Macular center | | | 21.42±5.81 | 21.44±5.74 | | 21.67±5.96 | 0.988 | | 0.845 | 0.853 |
| Inner | Nasal | | 34.35±5.98 | 34.96±6.14 | | 30.36±6.60 | 0.642 | | **0.004** | **0.001** |
|  | Superior | | 36.70±5.26 | 36.42±6.33 | | 33.88±7.17 | 0.059 | | **0.041** | 0.059 |
|  | Temporal | | 35.19±5.48 | 35.29±5.45 | | 31.90±6.06 | 0.929 | | **0.008** | **0.005** |
|  | Inferior | | 36.35±4.86 | 36.13±5.91 | | 33.86±6.04 | 0.850 | | **0.044** | 0.059 |
| Outer | Nasal | | 38.33±5.62 | 38.81±5.63 | | 38.07±5.39 | 0.677 | | 0.833 | 0.529 |
|  | Superior | | 37.33±4.63 | 37.38±5.77 | | 35.79±6.30 | 0.967 | | 0.208 | 0.182 |
|  | Temple | | 33.33±5.30 | 33.38±5.77 | | 31.60±5.45 | 0.966 | | 0.151 | 0.129 |
|  | Inferior | | 39.07±5.41 | 38.00±5.22 | | 36.45±5.84 | 0.355 | | **0.030** | 0.184 |
| **DVC** | |  | | |  | | |  | | |
| Average | | | 33.83±8.56 | 36.46±3.73 | | 34.18±6.71 | 0.058 | | 0.806 | 0.102 |
| Macular center | | | 19.67±6.78 | 21.40±5.60 | | 23.24±7.11 | 0.209 | | **0.013** | 0.181 |
| Inner | Nasal | | 35.40±9.17 | 38.17±4.36 | | 35.43±7.41 | 0.068 | | 0.983 | **0.037** |
|  | Superior | | 36.86±9.38 | 39.94±4.25 | | 36.48±8.78 | 0.059 | | 0.818 | **0.035** |
|  | Temporal | | 36.14±9.41 | 38.21±4.31 | | 36.55±7.35 | 0.176 | | 0.795 | 0.279 |
|  | Inferior | | 36.51±9.25 | 39.04±4.23 | | 36.38±7.95 | 0.104 | | 0.935 | 0.089 |
| Outer | Nasal | | 35.30±10.15 | 38.69±5.15 | | 35.19±7.74 | **0.042** | | 0.948 | **0.037** |
|  | Superior | | 34.63±9.44 | 37.67±5.33 | | 33.64±9.08 | 0.075 | | 0.574 | **0.020** |
|  | Temple | | 35.02±9.85 | 37.71±4.92 | | 35.57±7.25 | 0.092 | | 0.738 | **0.037** |
|  | Inferior | | 34.95±10.13 | 37.31±5.28 | | 35.17±6.94 | 0.144 | | 0.898 | 0.186 |

Values for continuous variables are means ± standard deviations for all subjects in each group.

Boldface values indicate statistically significant differences at *P* < 0.05.

DM, Diabetic patients without diabetic retinopathy; DR, Non-proliferative diabetic retinopathy; HM, Diabetic patients with high myopia.

*P*1: comparison between the group DM and DR; *P*2: comparison between the group DM and HM; *P*3: comparison between the group DR and HM.

* One-way ANOVA followed by the post hoc LSD test.

SVC, superficial vascular complex

DVC, deep vascular complex

RVD, vascular density of retinal
